# Lung ultrasound response to awake prone positioning predicts the need for intubation in patients with COVID-19 induced acute hypoxemic respiratory failure: an observational study

**DOI:** 10.1186/s13054-022-04064-3

**Published:** 2022-06-27

**Authors:** Miguel Ibarra-Estrada, María J. Gamero-Rodríguez, Marina García-de-Acilu, Oriol Roca, Laura Sandoval-Plascencia, Guadalupe Aguirre-Avalos, Roxana García-Salcido, Sara A. Aguirre-Díaz, David L. Vines, Sara Mirza, Ramandeep Kaur, Tyler Weiss, Claude Guerin, Jie Li

**Affiliations:** 1 Unidad de Terapia Intensiva, Hospital Civil Fray Antonio Alcalde, Universidad de Guadalajara, Guadalajara, Jalisco México; 2 Asociación Mexicana de Ultrasonografía Crítica y Urgencias, Guadalajara, México; 3 Servei de Medicina Intensiva, Parc Taulí Hospital Universitari, Sabadell, Spain; 4 Centro de Investigación Biomédica en Red de Enfermedades Respiratorias (CIBERES), Madrid, Spain; 5 Servicio de Pediatría, Hospital Civil “Juan I. Menchaca”, Guadalajara, Jalisco México; 6 Departamento de Infectología, Hospital Civil Fray Antonio Alcalde, Guadalajara, Jalisco México; 7 Department of Cardiopulmonary Sciences, Division of Respiratory Care, Rush University, Chicago, IL USA; 8 Department of Pulmonary and Critical Care Medicine, Rush University Medical Center, Chicago, IL USA; 9 Médecine Intensive Réanimation, Hôpital Édouard Herriot, Lyon, France; 10 Université de Lyon, Lyon, France; 11 Institut Mondor de Recherches Biomédicales, INSERM 955 CNRS 7000, Créteil, France

**Keywords:** COVID-19, Awake prone positioning, Lung ultrasound, High-flow nasal cannula, Intubation

## Abstract

**Background:**

Awake prone positioning (APP) reduces the intubation rate in COVID-19 patients treated by high-flow nasal cannula (HFNC). However, the lung aeration response to APP has not been addressed. We aimed to explore the lung aeration response to APP by lung ultrasound (LUS).

**Methods:**

This two-center, prospective, observational study enrolled patients with COVID-19-induced acute hypoxemic respiratory failure treated by HFNC and APP. LUS score was recorded 5–10 min before, 1 h after APP, and 5–10 min after supine in the first APP session within the first three days. The primary outcome was LUS score changes in the first three days. Secondary outcomes included changes in SpO_2_/FiO_2_ ratio, respiratory rate and ROX index (SpO_2_/FiO_2_/respiratory rate) related to APP, and the rate of treatment success (patients who avoided intubation).

**Results:**

Seventy-one patients were enrolled. LUS score decreased from 20 (interquartile range [IQR] 19–24) to 19 (18–21) (*p* < 0.001) after the first APP session, and to 19 (18–21) (*p* < 0.001) after three days. Compared to patients with treatment failure (*n* = 20, 28%), LUS score reduction after the first three days in patients with treatment success (*n* = 51) was greater (− 2.6 [95% confidence intervals − 3.1 to − 2.0] vs 0 [− 1.2 to 1.2], *p* = 0.001). A decrease in dorsal LUS score > 1 after the first APP session was associated with decreased risk for intubation (Relative risk 0.25 [0.09–0.69]). APP daily duration was correlated with LUS score reduction in patients with treatment success, especially in dorsal lung zones (*r* =  − 0.76; *p* < 0.001).

**Conclusions:**

In patients with acute hypoxemic respiratory failure due to COVID-19 and treated by HFNC, APP reduced LUS score. The reduction in dorsal LUS scores after APP was associated with treatment success. The longer duration on APP was correlated with greater lung aeration.

*Trial registration* This study was prospectively registered on clinicaltrials.gov on April 22, 2021. Identification number NCT04855162.

**Supplementary Information:**

The online version contains supplementary material available at 10.1186/s13054-022-04064-3.

## Background

By favoring a more homogeneous distribution of tidal volume and inducing the recruitment of dorsal areas of lungs, prone positioning improves oxygenation, lung compliance, and ventilation/perfusion matching [1, 2]. In selected patients with acute respiratory distress syndrome (ARDS) on invasive mechanical ventilation, prone positioning has been shown to reduce mortality [3]. As a noninvasive and low-cost treatment, awake prone positioning (APP) has been extensively used in non-intubated patients with coronavirus disease (COVID-19) induced acute hypoxemic respiratory failure (AHRF) [4]. We recently found that APP reduced the intubation rate within 28 days of enrollment for COVID-19 patients treated by high-flow nasal cannula (HFNC) [5]. However, whether or not the better lung aeration with APP can explain the better patient outcome in terms of intubation needs has been poorly investigated.

Lung ultrasound (LUS), a noninvasive, radiation-free, and bedside-available imaging tool, has gained popularity for lung assessment in critically ill patients [6]. Several studies have shown that LUS is a valuable tool in assessing lung aeration response to prone positioning in intubated ARDS patients [7–11]. However, the ability of LUS to predict patient response to prone positioning was variable between studies [7–10]. More recently, LUS has been used to assess the response to APP in non-intubated patients with COVID-19. APP responders [12], who were defined by the increment of partial pressure of arterial oxygen/fraction of inspired oxygen [PaO_2_/F_I_O_2_] ≥ 20 mmHg, had a greater decrease in LUS score after 3 h of APP than non-responders. However, the included patients had mild-to-moderate AHRF, the patients’ response was investigated only after the first session, and the predictors of intubation were not assessed due to the small sample size and the low intubation rate.

In our previous cohort study of prone-positioning in intubated patients with COVID-19, we found that although most patients had an improvement in oxygenation on the first session of prone positioning, only those who survived had a significant response over the second and third sessions of prone positioning, which suggests that serial measurements along the first three days of treatment rather than the first session only, might improve the accuracy of prediction regarding patient-centered outcomes [13]. Moreover, in a large randomized controlled trial investigating the effects of APP on non-intubated patients with COVID-19, we found that LUS scores after three days of APP treatment decreased only in patients who eventually avoided intubation/death [14]. Therefore, we performed this study with two aims: 1) to explore the aeration response to APP in patients with AHRF induced by COVID-19 by using LUS within the first three days, and 2) to explore whether the changes in LUS associated with APP can predict the need for intubation. We hypothesized that better lung aeration with APP was associated with a reduction in intubation rate.

## Methods

### Study design

This prospective observational study was registered on clinicaltrials.gov (NCT04855162), and performed at two hospitals with IRB approval (Rush University Medical Center No. 21040601 and Comité de Ética en Investigación Hospital Civil Fray Antonio Alcalde No. HCG/CEI-0753/21). Due to the noninvasive nature of the assessment, written consent was waived by ethics committees at both hospitals.

### Patients

Consecutive patients ≥ 18 years with AHRF induced by COVID-19 confirmed by RT-PCR, treated by HFNC and APP were included. AHRF was defined by pulse oximetry (SpO_2_)/FiO_2_ < 315. Pregnant patients and those on palliative care or extracorporeal membrane oxygenation were excluded. Patients with APP ≤ 1 h/day or any missed LUS on the first APP session within the first three days of enrollment were withdrawn. Patients were encouraged to stay in the prone position for ≥ 8 h/day, as in our meta-trial, the treatment success was related to this threshold [5].

### Study procedures

LUS was performed by clinicians with > 8 years of clinical experience in LUS on critically-ill patients and certified trainers by WINFOCUS (*World Interactive Network Focused on Critical Ultrasound*). An Edge II (Fujifilm) or TE7 ultrasound system (Mindray, Shenzhen, China) with a curved transducer (3–8 MHz) was used. Depth was set at 6–10 cm according to patient size, with focus placed in pleural line, and gain regulated to optimize lung artifacts. The LUS evaluations were performed daily within the first three days of enrollment and on the morning of the 4th day before APP, as long as patients had not been intubated. The LUS investigation was done 5–10 min before APP (pre-APP), 1 h after APP (post-APP), and 5–10 min after returning to the supine position (post-supine) following the first prone session of these days. At the supine position, dorsal zones were scanned on a short transient lateral decubitus position. Global LUS aeration score was measured over 12 lung zones (ventral, lateral, and dorsal), with total scores ranging from 0 to 36, with higher scores indicating less lung aeration. We chose this aeration score rather than the re-aeration score (from − 5 to + 1) [15], because our study involved comparisons between groups in the pre-prone state more than once, with changes monitored over three days rather than a single APP session. Moreover, it is strongly correlated with tissue density assessed by CT scan and extravascular lung water assessed by transpulmonary thermodilution [15, 16]. Scores were interpreted and validated by two expert clinicians blinded to the LUS procedure. SpO_2_/FiO_2_ ratio, respiratory rate (RR), and ROX index ([SpO_2_/FiO_2_]/RR) [17, 18] were also recorded at the same time points of LUS assessment. Criteria for intubation were standardized and similar to our published meta-trial [5].

### Outcomes

The primary outcome was the change in global LUS score from pre-APP on day 1 to pre-APP on day 4. Secondary outcomes included changes in SpO_2_/FiO_2_ ratio, RR, and ROX index, as well as the differences in these variables between patients who were intubated (treatment failure) and those who avoided intubation (treatment success), and between responders and non-responders. Responders were defined as those patients whose SpO_2_/FiO_2_ ratio increased by ≥ 20% after the supine positioning of the first APP session. Predictors of treatment success were also explored.

### Sample size

Using a mean of pre-prone LUS score of 18.7 and standard deviation (SD) of 4.4 and post-supine LUS score at day 3 of 16.9 and SD of 4.6 [14], a confidence level (1-α) of 95% and power (1-ß) of 95%, the number of patients was 70. Considering an attrition rate of 5%, we calculated the total sample size as 74.

### Statistical analysis

The normality of distribution for continuous variables was assessed by Kolmogorov–Smirnov test, and presented as mean ± SD or as median and interquartile range (IQR). Repeated measures ANOVA or Friedman’s test was used to compare differences of the variables pre-APP, post-APP, and post-supine. Comparison of continuous variables between groups (treatment success vs treatment failure, responders vs non-responders) were conducted with Student’s t or Mann–Whitney U test, while ANCOVA test was used to compare variables between two groups at the same time points with Bonferroni adjustment for baseline covariates. Correlation coefficients were assessed with Spearman’s test. Categorical variables are presented as counts and proportions with 95% confidence intervals (95% CIs) and were compared by Chi-square or Fisher’s exact test. To explore if the LUS score was associated with treatment success, multivariate logistic regression models were performed with one covariate introduced at a time to avoid overfitting, including those with a *p*-value < 0.20 in the univariate comparisons between groups. The accuracy of different variables in predicting treatment success was assessed by calculating the area under the receiver operating characteristic (AUROC) curves. Two-sided *p* ≤ 0.05 was considered statistically significant. Statistical analysis was performed with MedCalc (MedCalc Software Ltd Ostend, Belgium. Version 20.1) and GraphPad Prism software (version 9.3.1).

## Results

### General characteristics of the enrolled patients

**Fig. 1:**
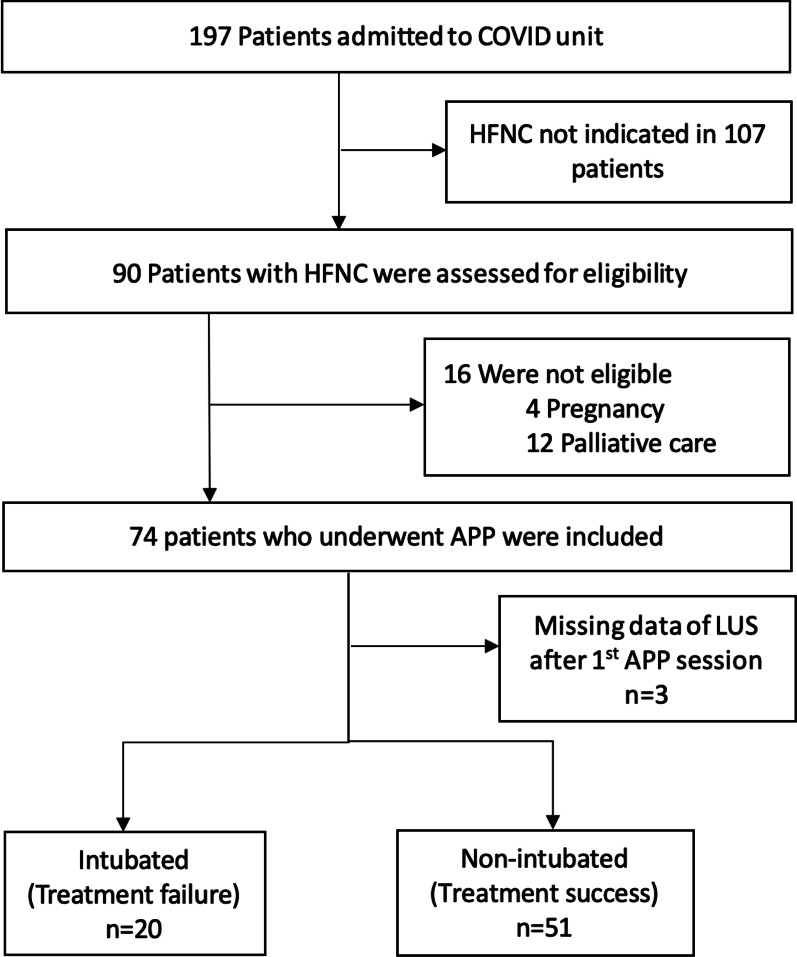
Flowchart of participants. *HFNC* high-flow nasal cannula; *LUS* lung ultrasound; *APP* awake prone positioning

From May 2, 2021, to August 29, 2021, 197 patients with COVID-19-induced AHRF were admitted to the two hospitals, of whom 90 were indicated for HFNC treatment. Sixteen were excluded, 12 due to palliative care and four due to pregnancy. In total, 74 patients with COVID-19 confirmed by RT-PCR and treated with HFNC and APP were included. All but one patient were recruited in general wards. Three did not tolerate APP ≥ 1 h on the second and third days, and were excluded for final analysis. Of the 71 patients, 20 (28.2%) had treatment failure (Fig. 1).

The median time from HFNC initiation to APP was 6 h (5.0–7.6), and the time from APP to intubation was 99 h (89–135). No patient was intubated within the first 72 h of HFNC. Except that patients with treatment success were younger than patients with treatment failure (40 [35–35] vs 46 [40–53] years, *p* = 0.01), no significant differences in baseline characteristics were found between the two groups (Table 1).

**Table 1:** Baseline characteristics and outcomes between patients with treatment failure and treatment success

Variable	Treatment failure (*n* = 20)	Treatment success (*n* = 51)	*p*
Age—years	46 (40–53)	40 (35–45)	0.01
Female sex—no. (%)	7 (35)	21 (41)	0.63
Body mass index^†^—kg/m^2^	30.9 (28.7–32.6)	30.4 (29.2–32.3)	0.81
Days from COVID confirmation	4.0 (2.5–6.0)	3.0 (2.0–4.0)	0.07
Comorbidities—no. (%)			
Pulmonary disease	3 (15)	1 (2)	0.06
Chronic kidney disease	3 (15)	2 (4)	0.10
Diabetes	3 (15)	3 (6)	0.21
Hypertension	4 (20)	3 (6)	0.07
Cardiovascular disease	0	5 (10)	0.31
Time from HFNC to APP initiation—hours	6.0 (5.0–7.0)	6.2 (5.0–7.8)	0.27
Before the first APP session			
Heart rate—beats/min	91 (82–99)	97 (87–105)	0.29
Respiratory rate—breaths/min	20 (18–23)	20 (18–22)	0.81
Mean arterial pressure—mmHg	85 (76–89)	82 (76–87)	0.30
SpO_2_^—^%	93 (91–96)	94 (92–97)	0.31
HFNC flow settings—L/min*	40 (40–40)	40 (40–40)	0.51
F_I_O_2_	1.0 (0.8–1.0)	0.9 (0.8–0.9)	0.28
SpO_2_:F_I_O_2_	107 (101–121)	92 (92–118)	0.09
ROX index	5.2 (4.3–6.2)	5.5 (4.7–6.6)	0.50
LUS score	20 (18–24)	20 (19–23)	0.74
Management			
Mean daily APP duration at first 3 days—hr/day	9.8 (8.9–12.1)	11.7 (8.7–16.5)	0.18
Dexamethasone—no. (%)	20 (100)	51 (100)	1.0
IL-6 modulators—no. (%)	4 (20)	6 (12)	0.45
Outcomes			
ICU length of stay—days	13 (9–16)	7 (6–9)	0.02
Hospital length of stay—days	19 (13–24)	11 (9–12)	< 0.001
Mortality—no. (%)	8 (40)	0	< 0.001

Medians with interquartile ranges are in parentheses. *APP* awake prone positioning; *HFNC* high-flow nasal cannula; *SpO*_*2*_ saturation of pulse oximetry; *F*_*I*_*O*_*2*_ fraction of inspired oxygen; *ROX* SpO_2_:F_I_O_2_/respiratory rate

^†^Body-mass index is the weight in kilograms divided by the square of the height in meters

*HFNC was provided using Precision Flow Hi-VNI™ (Vapotherm, Exeter, NH) with maximum flow of 40 L/min

### Lung aeration response to APP between patients with treatment success and treatment failure

**Fig. 2:**
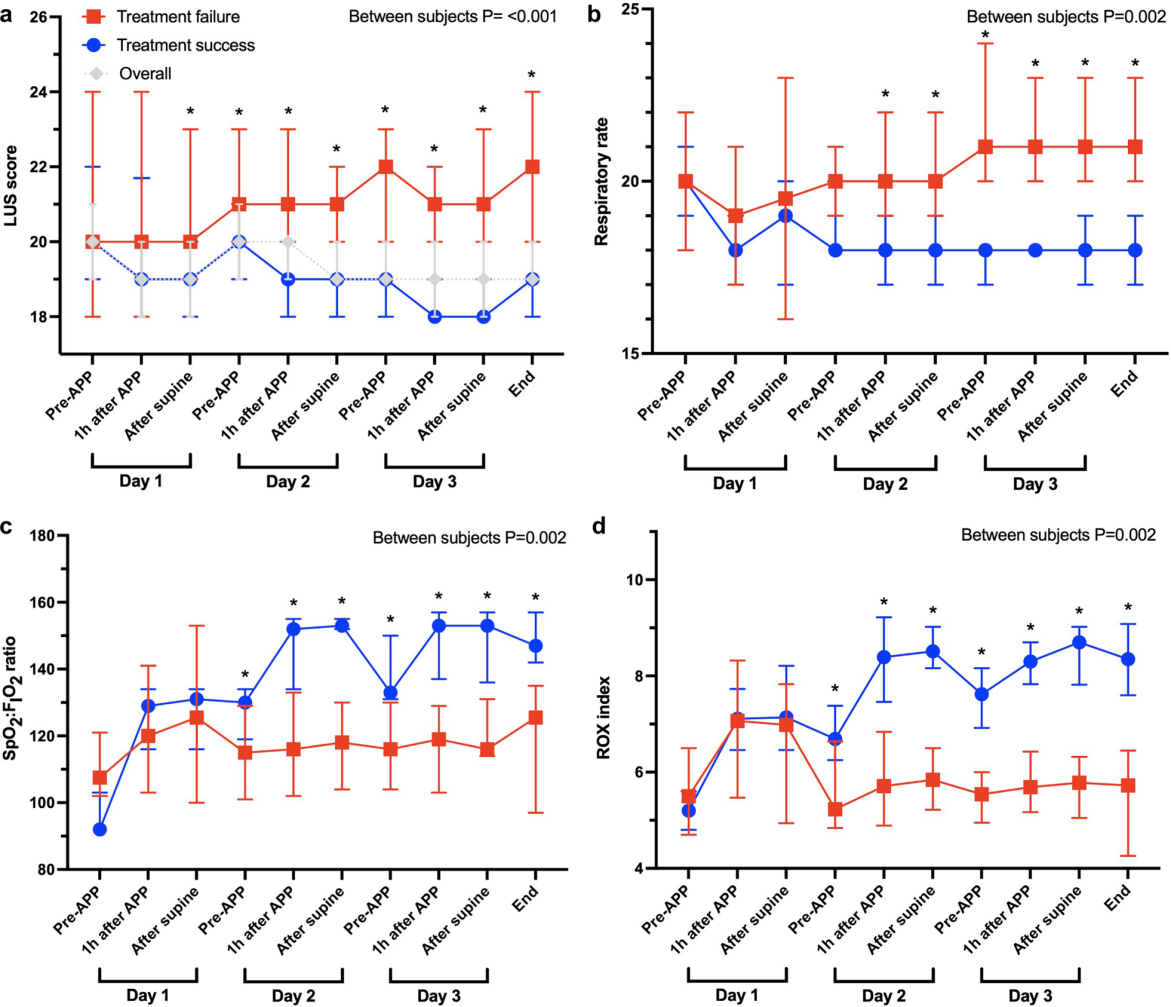
Evolution of measured variables. Lung ultrasound score (LUS) (**a**), respiratory rate (**b**), SpO_2_:F_I_O_2_ ratio (**c**), ROX index (**d**) between patients with treatment failure (red symbols and lines) and success (blue symbols and lines) and in the whole cohort (grey symbols and lines). *p < 0.05 between treatment failure and treatment success

On days 1, 2, and 3, the duration of the first APP sessions were 4.3 h (3.1–5.8), 4.0 h (3.5–4.6), and 4.3 h (3.5–5.1), respectively. LUS score decreased from 20.0 (19.0–24.0) pre-APP to 19.0 (18.0–21.0) (*p* < 0.001) post-supine at the first session, and to 19.0 (18.0–21.0) (*p* < 0.001) after three days. However, global LUS scores over the first three days were significantly reduced in patients with treatment success (from 20.0 [19.0–23.7] to 19.0 [18.0–20.0], *p* < 0.001), when no significant changes were observed in patients with treatment failure (Fig. 2a). More importantly, at the first APP session, patients with treatment success had a lower post-supine global LUS score than patients with treatment failure (19.0 [18.0–21.0] vs 20 [19.0–23.5], *p* = 0.01), even though they had a similar pre-APP global LUS score (*p* = 0.74).

**Fig. 3:**
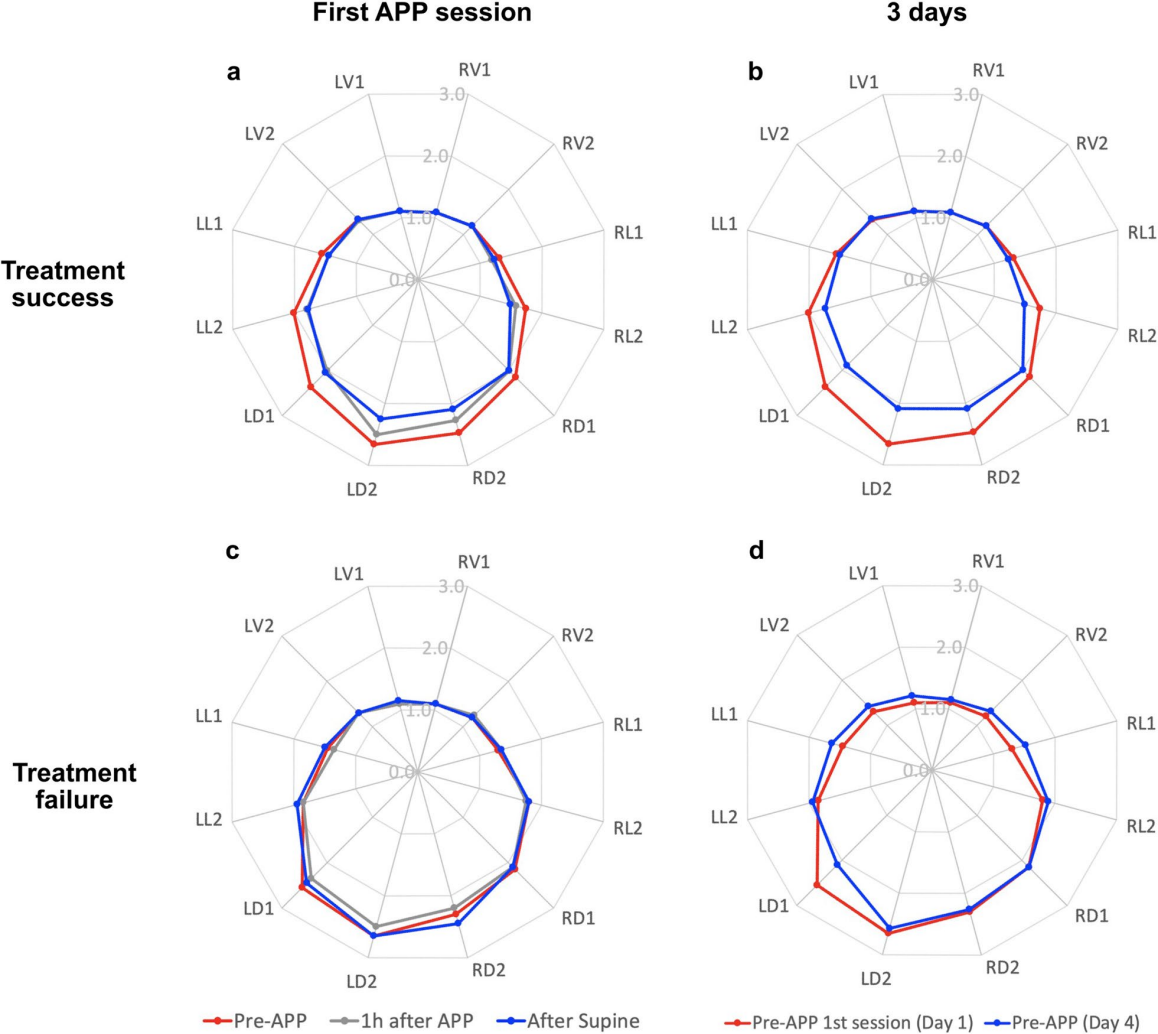
Response in regional lung ultrasound (LUS) score to the first APP session on the first day and the first three days. Radar plots show the response in LUS score for patients with treatment success to the first session of APP and after three days. Each axis of the plots represents LUS score of a single lung zone, with the response observed as the change from red (before APP) to gray (1 h after APP) and blue (after supine) lines. For patients with treatment success, LUS score in both dorsal lung zones (LD1, LD2, RD1, and RD2) and some lateral zones (LL2 and RL2) decreased at the first session of APP (**a**) and after three days (**b**). While for patients with treatment failure, LUS score did not change in all lung zones at the first session of APP (**c**) and after three days (**d**), except for LD1 after three days. *RV* right ventral; *RL* right lateral; *RD* right dorsal; *LV* left ventral; *LL* left lateral; *LD* left dorsal; *APP* awake prone positioning; *LUS* lung ultrasound

**Fig. 4:**
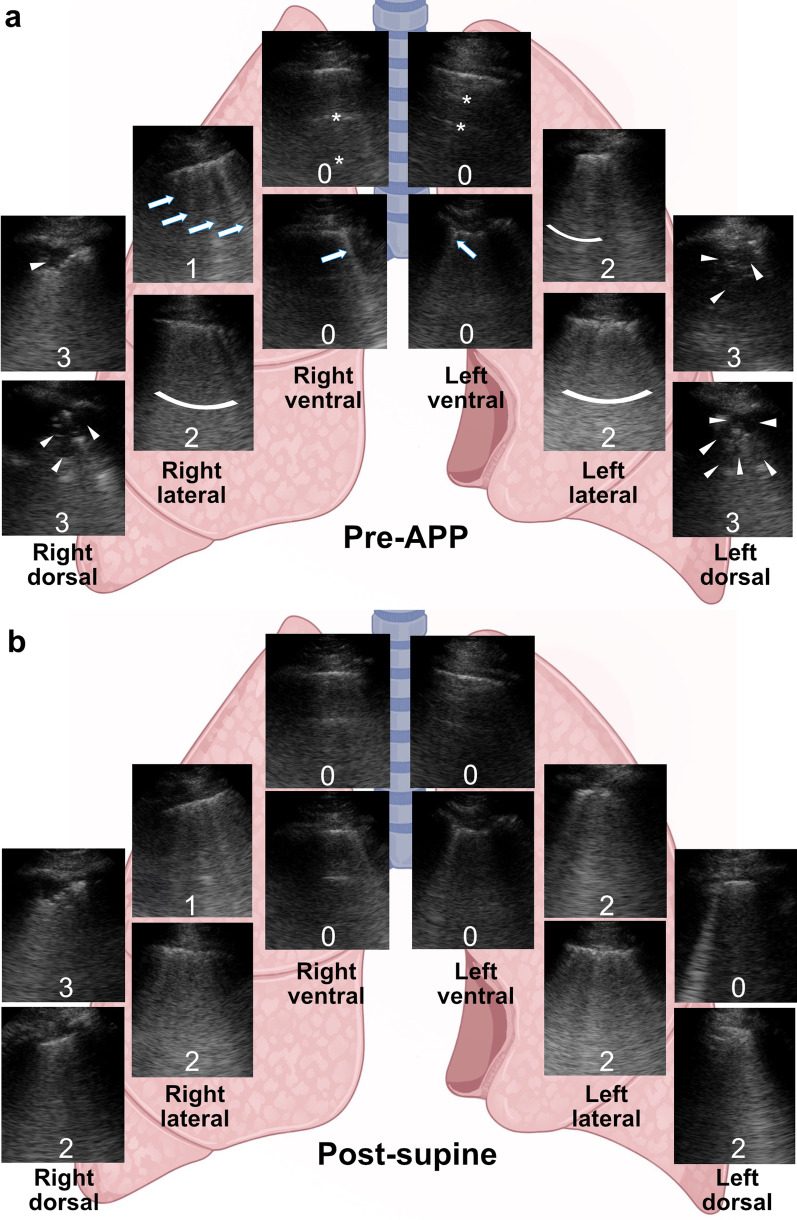
Typical changes in LUS score to APP in a patient with treatment success. **a** The patient’s LUS score at each lung zone before APP. Ventral zones had predominantly A-lines (asterisks) and ≤ 2 B-lines (arrows) (0 point) meaning normal aeration; lateral zones had ≥ 3 well-spaced B-lines (arrows) (1 point) and coalescent B-lines (curved bars) (2 points) suggesting moderate and severe aeration loss, respectively; and dorsal zones had irregular pleura, tissue-like pattern and subpleural consolidations (arrowheads) (3 points) suggesting complete loss of aeration. **b** The patient’s LUS score at each lung zone after returning to the supine position. Ventral, lateral, and upper right dorsal zones remained unchanged, while upper left dorsal zone improved from complete loss to normal aeration (3 to 0 points), and lower dorsal zones improved from complete to severe loss of aeration bilaterally (3 to 2 points). Total LUS score decreased from 19 to 14 in this patient whose first APP session lasted 5.5 h. The patient was in the supine position when LUS was performed. LUS, lung ultrasound; APP, awake prone positioning

Over the 12 LUS zones, the response in aeration was characterized by a pattern of bilateral improvement in the dorsal lung from consolidation (3 points) to severe loss of aeration defined by coalescent B lines (2 points) (Fig. 3). At the first APP session, dorsal LUS score in both lungs significantly decreased in patients with treatment success (from 10.0 [8.0–11.0] pre-APP to 8.0 [8.0–9.0] post-supine, *p* < 0.001) (Fig. 3a), versus no changes in patients with treatment failure (Fig. 3c). Similarly, after the first three days, the dorsal LUS score in both lungs was significantly reduced in patients with treatment success (from 10.0 [8.0–11.0] to 8.0 [7.0–8.0], *p* < 0.001) (Fig. 3b), with no change in patients with treatment failure (Fig. 3d and see Additional file 1: Table S1). The number of patients with a score of 3 at any dorsal zone pre-APP was similar in groups of treatment success and treatment failure (67% vs 70%, *p* = 0.78); however, compared to the treatment failure group, more patients in the treatment success group had an improvement from 3 to 2 (65% vs 35%, *p* = 0.03) and from 3 to 1 (29% vs 5%, *p* = 0.03). Ventral LUS score increased from 1 to 2 in 3 patients (15%) in the treatment failure group, compared to 3 patients (6%) in the treatment success group (*p* = 0.34). Typical changes in a patient with treatment success are depicted in Fig. 4. Regional changes after APP sessions on days 2 and 3 were similar between treatment success and failure groups (Additional file 1: Fig. S1).

Responders to APP in terms of oxygenation had a similar reduction in global LUS score as non-responders following the first APP session (between subjects *p* = 0.07) (see Additional file 1: Fig. S2a). However, when considering only the dorsal LUS score, compared to pre-APP, responders had a greater reduction than non-responders at post-supine following the first APP session (-1.1 [-1.6 to − 0.5] vs − 0.6 [-1.1 to − 0.2], between subjects *p* = 0.01) (see Additional file 1: Fig. S2b).

### Changes in LUS scores and mean daily APP duration

The total duration of APP on days 1, 2, and 3 were 10.7 h (8.3–15.9), 10.0 h (IQR 8.4–15.7), and 11.6 h (9.4–16.0), respectively. The mean daily duration of APP within the first three days significantly correlated to a decrease in global LUS score during the same timeframe (*r* = -0.65; *p* < 0.001), but not to SpO_2_/F_I_O_2_ ratio (*r* = 0.03; *p* = 0.82) or ROX index (*r* = 0.15; *p* = 0.23) (see Additional file 1: Fig. S3). Specifically, only the response in dorsal lung zones significantly correlated with the mean daily duration of APP in patients with treatment success (*r* = -0.76; *p* < 0.001) (see Additional file 1: Fig. S4).

### Other physiological responses to APP

At 1 h of APP, SpO_2_/F_I_O_2_ ratios, and ROX indexes were significantly improved on all three days for all 71 patients (all *p* < 0.05) (see Additional file 1: Fig. S5), while these responses were maintained upon return to supine. The correlation coefficients of APP response between global LUS scores and SpO_2_/F_I_O_2_ ratios were *r* = 0.25 (*p* = 0.03), *r* = 0.05 (*p* = 0.70), and *r* = 0.24 (*p* = 0.04) on the first, second and third day, respectively. However, no significant correlations between changes in global LUS score and ROX index or RR in the first APP sessions of the first three days were observed (see Additional file 1: Fig. S6).

In both groups of patients with treatment success and treatment failure, RR (Fig. 2b), SpO_2_/F_I_O_2_ ratio (Fig. 2c), and ROX index (Fig. 2d) were all significantly improved at post-supine in the first APP session (all *p* < 0.05), and the increments in two groups were similar. However, compared to patients with treatment failure, more patients with treatment success met the oxygenation response criteria (30% vs 57%, *p* = 0.04). Moreover, only patients with treatment success showed a significant improvement in SpO_2_/F_I_O_2_ ratio and ROX index post-supine on the second and third days and RR on the second day. Overall improvement of RR, SpO_2_/F_I_O_2_ ratio, and ROX index in the three days were greater in patients with treatment success (all *p* < 0.05).

### Prediction of treatment success among patients who received APP during HFNC treatment

A decrease in dorsal LUS score > 1 at post-supine in the first APP session had the highest area under the curve for predicting treatment success (0.75 [0.69–0.88]), with a sensitivity of 80%, specificity of 60% (Table 2 and see Additional file 1: Fig. S7), and a calculated relative risk of 0.25 (0.09–0.69) for being intubated. This predictor was constantly associated with a decreased risk of intubation regardless of multiple adjustments for other relevant covariates (see Additional file 1: Table S2).

**Table 2:** ROC curve analyses of responses in terms of lung aeration at each observed session for prediction of treatment success

Variable	AUC (95% CI)	*p*	Cut-off	Sens	Spec	LR +	LR—
Day 1							
Δ-Dorsal LUS	0.75 (0.69–0.88)	< 0.001	< − 1	80	60	2.04	0.33
Δ-ROX index	0.65 (0.52–0.76)	0.05	> 0.5	55	88	4.67	0.51
Δ-RR	0.53 (0.41–0.65)	0.67	< − 1	40	72	1.46	0.83
Δ-SpO_2_/FiO_2_ ratio	0.65 (0.53–0.76)	0.04	> 7	50	84	3.19	0.59
Day 2							
Δ-Dorsal LUS	0.63 (0.50–0.74)	0.07	< − 2	78	43	1.39	0.49
Δ-ROX index	0.75 (0.63–0.84)	< 0.001	> 1.2	89	54	1.98	0.19
Δ-RR	0.66 (0.53–0.77)	0.02	< − 1	63	64	1.79	0.57
Δ-SpO_2_/FiO_2_ ratio	0.66 (0.53–0.77)	0.03	> 1.1	47	86	3.45	0.61
Day 3							
Δ-Dorsal LUS	0.51 (0.39–0.63)	0.86	< − 2	15	90	1.61	0.93
Δ-ROX index	0.57 (0.44–0.69)	0.32	> 1.8	100	23	1.31	0
Δ-RR	0.67 (0.54–0.77)	0.01	< − 2	26	98	13.4	0.75
Δ-SpO_2_/FiO_2_ ratio	0.60 (0.48–0.72)	0.12	> 15	89	39	1.47	0.27
Whole 3 days							
Δ-Dorsal LUS	0.72 (0.62–0.84)	0.005	< − 1	50	82	2.83	0.61
Δ-ROX index	0.84 (0.73–0.92)	< 0.001	> 0.3	50	90	5.10	0.55
Δ-RR	0.71 (0.59–0.82)	0.005	< 0	57	84	3.69	0.50
Δ-SpO_2_/FiO_2_ ratio	0.77 (0.64–0.86)	< 0.001	> 42	100	51	2.04	0

*Δ-Dorsal LUS* change in lung ultrasound score after the APP session; *Δ-ROX* change in ROX index after the APP session; *Δ-RR* change in respiratory rate after the APP session; *Δ-SpO*_*2*_*/FiO*_*2*_* ratio* change in SpO_2_/FiO_2_ ratio after the APP session; *AUC* area under the curve; *Sens* sensitivity; *Spec* specificity; *LR* + positive likelihood ratio; *LR* − negative likelihood ratio

## Discussion

In this study among patients with COVID-19-induced AHRF, we found that: 1) compared to patients with treatment failure, those with treatment success had a greater reduction in dorsal LUS score post-supine following the first APP session, 2) changes of RR, ROX index, SpO_2_/F_I_O_2_ after the first APP session did not differ between patients with treatment success vs. failure, 3) a reduction in the dorsal LUS score > 1 after the first APP session was independently associated with a lower risk of treatment failure, and 4) mean daily APP duration was significantly correlated to a decrease in global LUS score, and particularly to the reduction in dorsal LUS score for patients with treatment success.

Franchineau and colleagues reported time-dependent effects of prone positioning on lung recruitment assessed by electrical impedance tomography (EIT) in intubated ARDS patients on veno-venous ECMO, especially in dorsal zones [19]. This time-dependent improvement in the aeration of the dorsal zones may lead to a more homogeneous distribution of lung inflation, improving lung compliance, decreasing the inspiratory effort [20], and providing a more protective distribution of stress and strain.

Furthermore, we also found that the reduction in dorsal LUS score > 1 in the first APP session predicted the treatment success. Importantly, this finding can help early identify those patients with high risk of APP failure, who might benefit from more intensive monitoring and early treatment escalation and not delay intubation [21]. Thus, LUS assessment may be a very useful tool in the day-to-day clinical decision-making process.

In contrast to the correlation between the changes in global LUS score and the APP duration, the duration of APP was not correlated with the changes in oxygenation. This may be explained by the fact that prone-related oxygenation response is determined by the balance between the resolution of dorsal atelectasis and the formation of ventral atelectasis on the one hand, and the changes in lung perfusion, on the other hand [22]. It is worth noting that in 35% of intubated patients in the study by Rossi et al., oxygenation decreased after prone positioning. They explained these negative changes by the prevalence of consolidated lung tissue, which was less likely to be recruited with prone positioning [22]. Therefore, using the oxygenation response to assess the extent of dorsal recruitment during prone positioning might not be appropriate. Fossali et al. found similar results in a recent physiological study, where they evaluated 21 intubated patients with COVID-19 with CT scan and EIT [23]. They found extensive alveolar recruitment in dorsal regions with prone positioning, along with alveolar derecruitment in ventral zones, which was, however, to a smaller extent than recruitment in the dorsal regions. No significant correlation between global or regional lung recruitment and oxygenation was found [23]. Additionally, given that LUS can assess the severity of COVID-19 pneumonia as good as the chest CT scans in most symptomatic patients with confirmed COVID-19 [24], our results support the utility of LUS to assess the lung morphology and monitor its changes with APP, especially if CT scan is not available or in patients at high risk for moving to the CT scan room. Moreover, we found that more patients in the treatment success group had improvement in dorsal zone LUS scores from 3 to 2 (consolidation to coalescent B lines) than in the treatment failure group. As we did not perform an extended LUS, we cannot exclude that this finding could be due to the existence of different pathologies (atelectasis vs pneumonia) [25].

Prone positioning significantly improved oxygenation in the first pronation for both patients with treatment failure and success. However, oxygenation improvement associated with prone positioning persisted on the second and third days only in those patients with treatment success. Similar results were obtained in previous studies on non-intubated [26] and intubated [13] COVID-19 patients who received prone positioning. However, our previous study with non-intubated patients with COVID-19 found that patient oxygenation response to APP on the second day could predict the need for intubation [13], while this was not significant in the current study. The discrepancy might be explained by the smaller sample size (71 vs 108) and the greater severity (average SpO_2_/F_I_O_2_ of 100 vs 150 at the study enrollment). In contrast, patient response to the first APP session assessed by LUS was significantly associated with the treatment success, implying that LUS response to the first APP session was more sensitive than other physiological parameters such as oxygenation responses to APP in predicting the need for intubation.

This study has several strengths. First, to our knowledge, this is the first to report the predictive value of global LUS scores for intubation in COVID-19 patients receiving APP. Second, this is a two-center study with a large and predefined sample size to assess patient response to APP using LUS. Third, a blinded interpretation of the LUS was performed by two expert clinicians. Lastly, we assessed LUS scores in the first APP sessions on three consecutive days rather than a single session.

This study also has several limitations. First, the LUS assessment was performed only once daily, whether the APP response in the other sessions would have been different was unknown. Second, we did not evaluate other ultrasonographic variables such as diaphragm displacement or thickening fraction that were utilized to assess inspiratory effort during APP for COVID-19 patients [27]. Third, the contribution of HFNC added to APP in the recruitment of the dorsal regions might be different from the combined use of noninvasive ventilation or conventional oxygen with APP, so our findings could not be generalized to patients treated with other noninvasive respiratory support therapies. Lastly, this study was designed to investigate the change in global LUS score after 3 days of APP in a single group, therefore, the results regarding predictors of intubation should be validated in another study that incorporates a control group of patients who will remain in the supine position, which might raise an ethical issue.

## Conclusion

For patients with COVID-19-induced AHRF and treated by HFNC and APP, APP was associated with a time-dependent improvement of the aeration of the dorsal lung zones. On the supine position after the first APP session, patients with treatment success had a greater reduction in the dorsal LUS score than patients with treatment failure. This reduction predicted the avoidance of intubation, helping to early identify those patients with a higher risk of intubation. LUS assessment is an easy-to-use tool that facilitates bedside clinical decision-making for critically ill patients treated with HFNC who require APP.

## Supplementary Information

**Additional file 1**: **Table S1**. Values of LUS scores at the first APP session and at the 3 days of observation according to groups. **Figure S1.** Responses in LUS score, SpO_2_:F_I_O_2_ ratio, ROX index and respiratory rate at the APP sessions. **Figure S2.** Response in global and dorsal LUS score after the first APP session. **Figure S3.** Correlation of mean daily duration of APP of the first 3 days with response in overall LUS score, respiratory rate SpO_2_:F_I_O_2_ ratio, and ROX index. **Figure S4.** Mean daily duration of APP at 3 days and LUS score changes in dorsal and ventral lung zones. **Figure S5.** Responses in SpO_2_:F_I_O_2_ ratio, ROX index, and respiratory rates at the first morning APP sessions of the 1st, 2nd and 3rd days. **Figure S6.** Correlation between response in LUS score with SpO_2_:F_I_O_2_ ratio, ROX index and respiratory rate at the first morning APP sessions of the 1st, 2nd and 3rd days. **Figure S7.** Comparison between areas under the ROC curves of responses to APP at the first session. **Table S2.** Logistic regression to analyze the effect of ≥ 1 point decrease in dorsal LUS score at the first APP on the risk of intubation.

## Data Availability

After publication, de-identified data will be available for sharing upon reasonable requests to the corresponding author made by researchers.

## Abbreviations

APP: Awake prone positioning
HFNC: High-flow nasal cannula
LUS: Lung ultrasound
COVID-19: Coronavirus disease
AHRF: Acute hypoxemic respiratory failure
IQR: Interquartile range
ARDS: Acute respiratory distress syndrome
PaO_2_/F_I_O_2_: Partial pressure of arterial oxygen/fraction of inspired oxygen
IRB: Institutional review board
SpO_2_: Oxygen saturation of pulse oximetry
FiO_2_: Fraction of inspired oxygen
WINFOCUS: World Interative Network Focused on Critical Ultrasound
SD: Standard deviation
ANOVA: Analysis of variance
ANCOVA: Analysis of covariance
CI: Confidence interval
AUROC: Area under the receiver operating characteristic
EIT: Electrical impedance tomography
ECMO: Extracorporeal membrane oxygenation
CT: Computed tomography
