# Supplementary material for: Lung ultrasound response to awake prone positioning predicts the need for intubation in patients with COVID-19 induced acute hypoxemic respiratory failure: an observational study
Source: Crit Care. 2022 Jun 27;26:189. doi: 10.1186/s13054-022-04064-3 (PMC9235111; doi:10.1186/s13054-022-04064-3)
Supplement: Supplementary file 1 — Additional file 1: Table S1. Values of LUS scores at the first APP session and at the 3 days of observation according to groups. Figure S1. Responses in LUS score, SpO2:FIO2 ratio, ROX index and respiratory rate at the APP sessions. Figure S2. Response in global and dorsal LUS score after the first APP session. Figure S3. Correlation of mean daily duration of APP of the first 3 days with response in overall LUS score, respiratory rate SpO2:FIO2 ratio, and ROX index. Figure S4. Mean daily duration of APP at 3 days and LUS score changes in dorsal and ventral lung zones. Figure S5. Responses in SpO2:FIO2 ratio, ROX index, and respiratory rates at the first morning APP sessions of the 1st, 2nd and 3rd days. Figure S6. Correlation between response in LUS score with SpO2:FIO2 ratio, ROX index and respiratory rate at the first morning APP sessions of the 1st, 2nd and 3rd days. Figure S7. Comparison between areas under the ROC curves of responses to APP at the first session. Table S2. Logistic regression to analyze the effect of ≥ 1 point decrease in dorsal LUS score at the first APP on the risk of intubation. [file 13054_2022_4064_MOESM1_ESM.pdf]

Supplementary Appendix

Supplement to: Ibarra-Estrada M, Gamero-Rodríguez M, García-de-Acilu M, et al. Lung Ultrasound Response to Awake Prone Positioning Predicts Intubation in COVID-19.

Table of contents:

**Table S1**. Values of LUS scores at the first APP session and at the 3 days of observation according to groups.	Page 2
**Figure S1.** Responses in LUS score (**a, b, c**), SpO_2:F_IO_2 ratio (**d, e, f**), ROX index (**g, h, i**) and respiratory rate (**j, k, l**) at the APP sessions.	Page 3
**Figure S2.** Response in global and dorsal LUS score after the first APP session.	Page 4
**Figure S3.** Correlation of mean daily duration of APP of the first 3 days with response in overa LUS score (**a**), respiratory rate (**b)** SpO_2:F_IO_2 ratio (**c**), and ROX index (**d**).	Page 5
**Figure S4.** Mean daily duration of APP at 3 days and LUS score changes in dorsal and ventral lung zones.	Page 6
**Figure S5.** Responses in SpO_2:F_IO_2 ratio (**a, b, c**), ROX index (**d, e, f**), and respiratory rates at the first morning APP sessions of the 1st, 2nd and 3rd days.	Page 7
**Figure S6.** Correlation between response in LUS score with SpO_2:F_IO_2 ratio, ROX index and respiratory rate at the first morning APP sessions of the 1st (**a, b, c**), 2nd (**d, e , f**) and 3rd (**g, h, i**) days.	Page 8
**Figure S7.** Comparison between areas under the ROC curves of responses to APP at the first session.	Page 9
**Table S2.** Logistic regression to analyze the effect of ≥1 point decrease in dorsal LUS score at the first APP on the risk of intubation.	Page 10

Table S1. Values of LUS scores at the first APP session and at the 3 days of observation according to groups

	First APP session			Whole 3 days
	Pre-APP	1-h on APP	After APP	Pre-APP
Treatment success	20 (19-24)	19 (18-21)	19 (18-21)	19 (18-20)
Treatment failure	20 (18-24)	20 (18-24)	20 (19-23)	22 (20-23)

Figure S1. Response in regional LUS score after the first APP sessions of days 2 and 3.

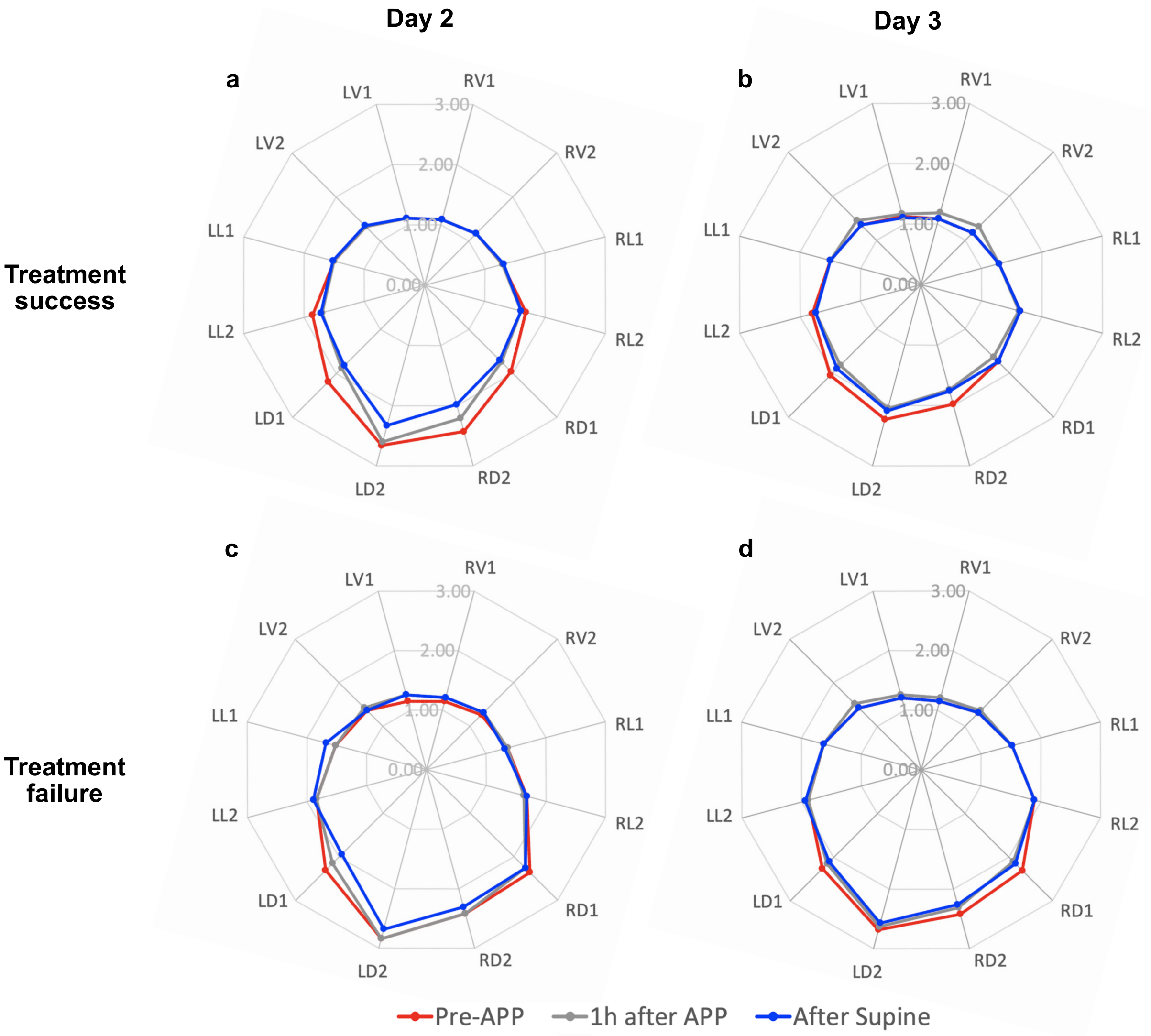

Patients with treatment success at days 2 (**a**) and 3 (**b**). Patients with treatment failure at days 2 (**c**) and 3 (**d**). RV, right ventral; RL, right lateral; RD, right dorsal; LV, left ventral; LL, left lateral; LD, left dorsal; LUS, lung ultrasound score; APP, awake prone positioning.

Figure S2. Response in global and dorsal LUS score after the first APP session. Between subject P= 0.07 for global score and P=0.02 for dorsal score.
LUS, lung ultrasound; APP, awake prone positioning. *P<0.05, compared to responders.

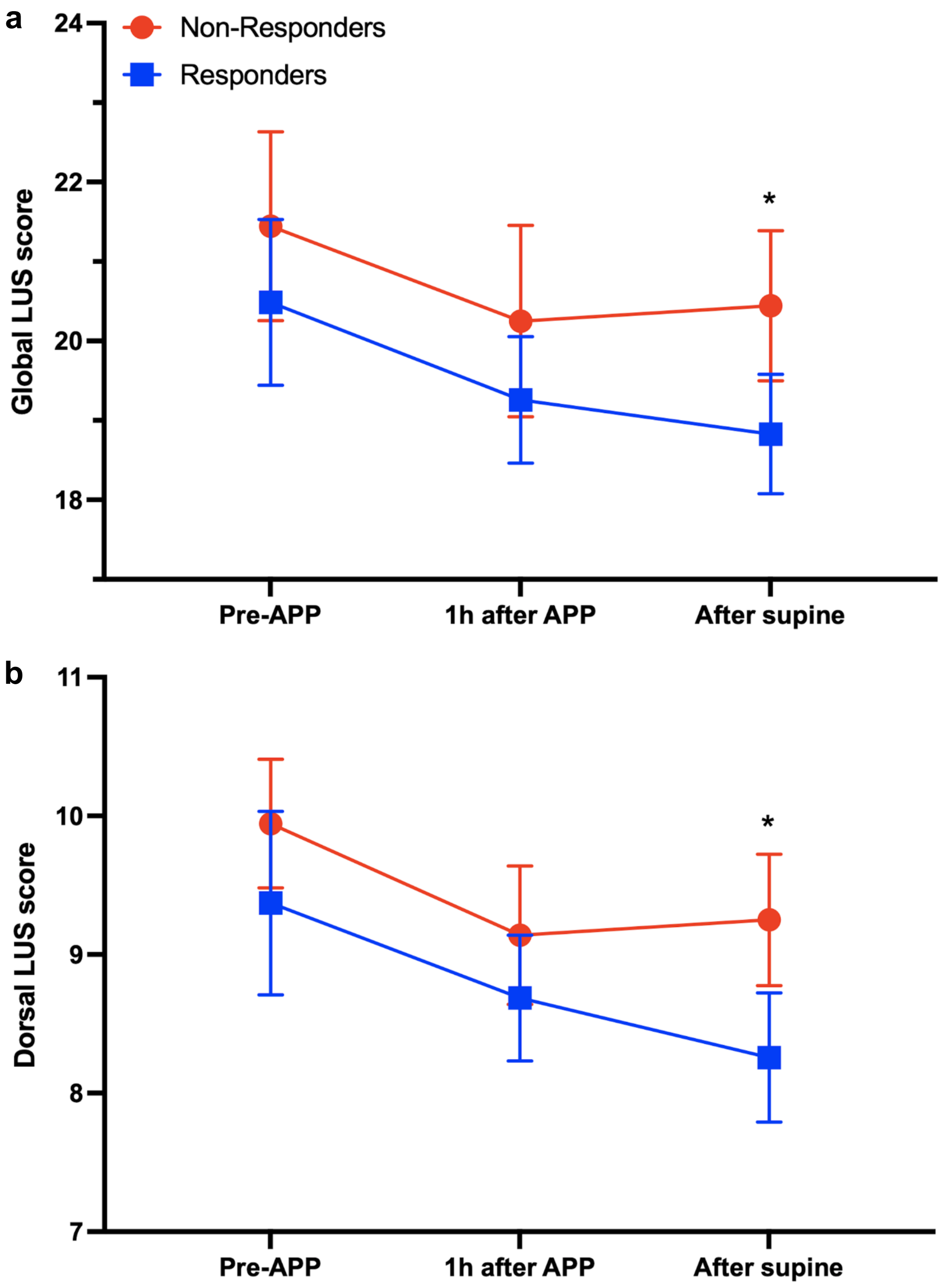

Figure S3. Correlation of mean daily duration of APP of the first 3 days with response in overall LUS score (**a**), respiratory rate (**b)** SpO_2:F_IO_2 ratio (**c**), and ROX index (**d**). Red and blue dots correspond to treatment failure and treatment success patients, respectively. LUS, lung ultrasound. APP, awake prone positioning.

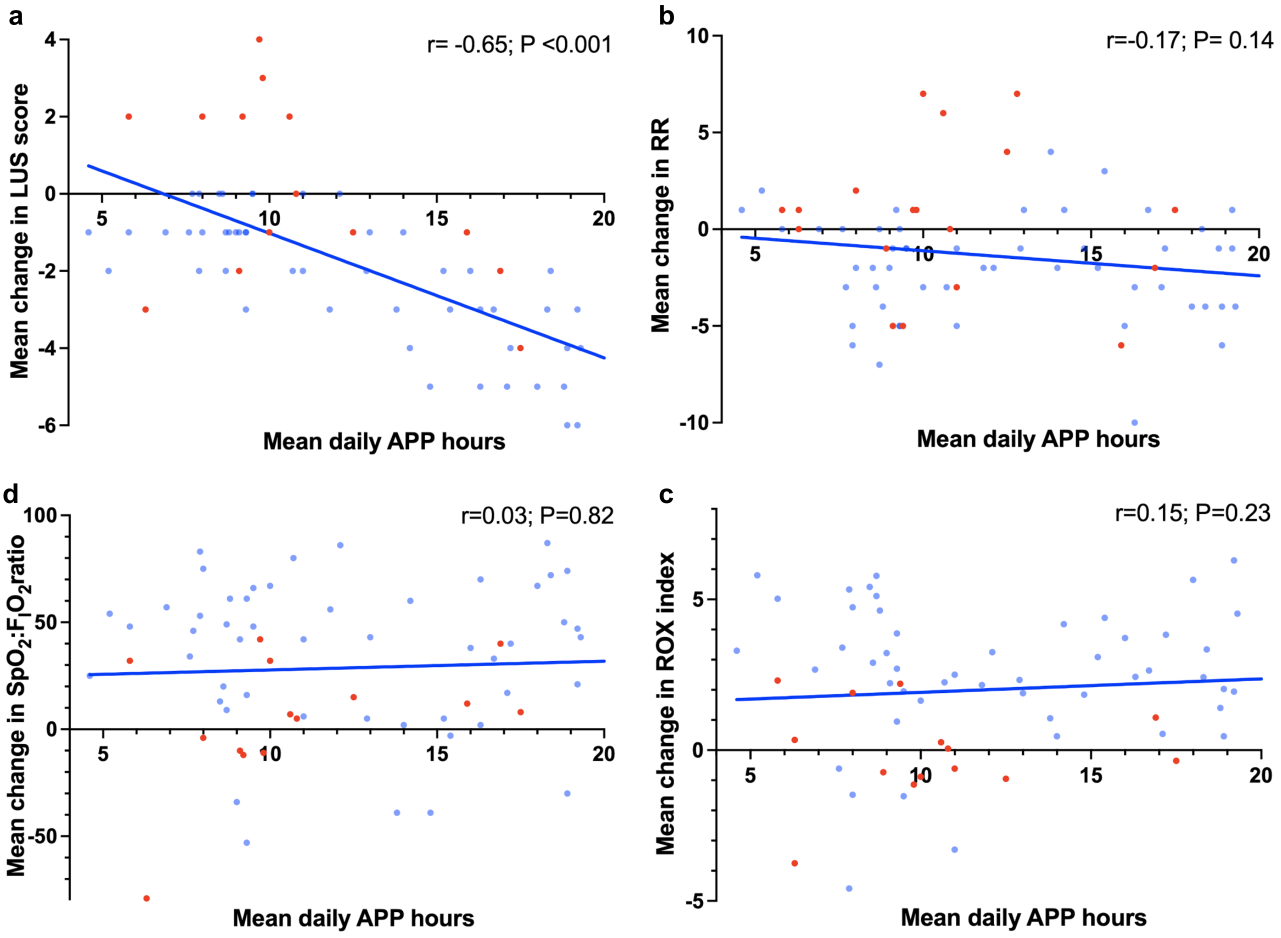

Figure S4. Mean daily duration of APP at 3 days and LUS score changes in dorsal and ventral lung zones. LUS, lung ultrasound; APP, awake prone positioning.

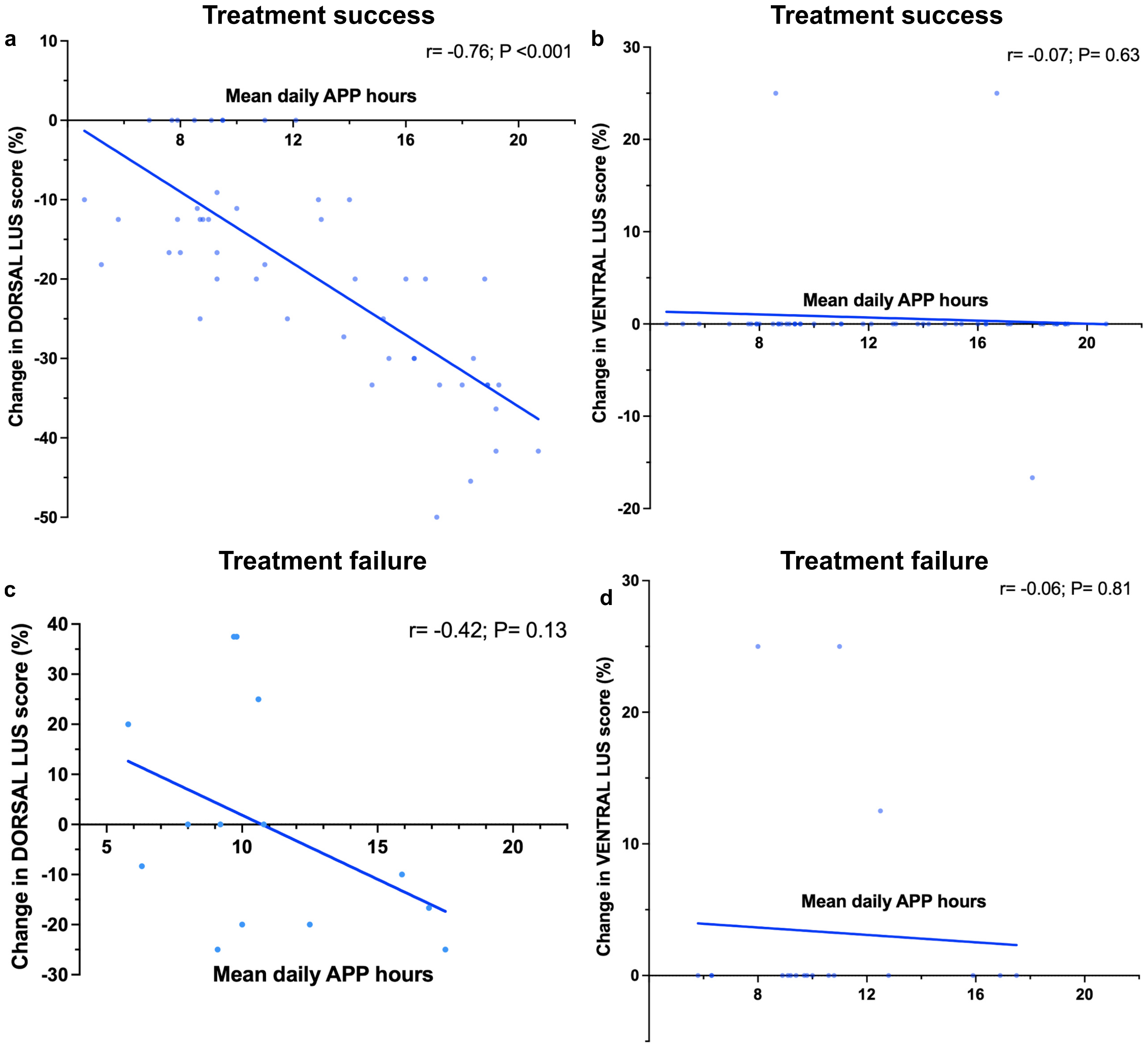

Figure S5. Responses in SpO2:FIO2 ratio (**a, b, c**), ROX index (**d, e, f**), and respiratory rates at the first morning APP sessions of the 1st, 2nd and 3rd days.

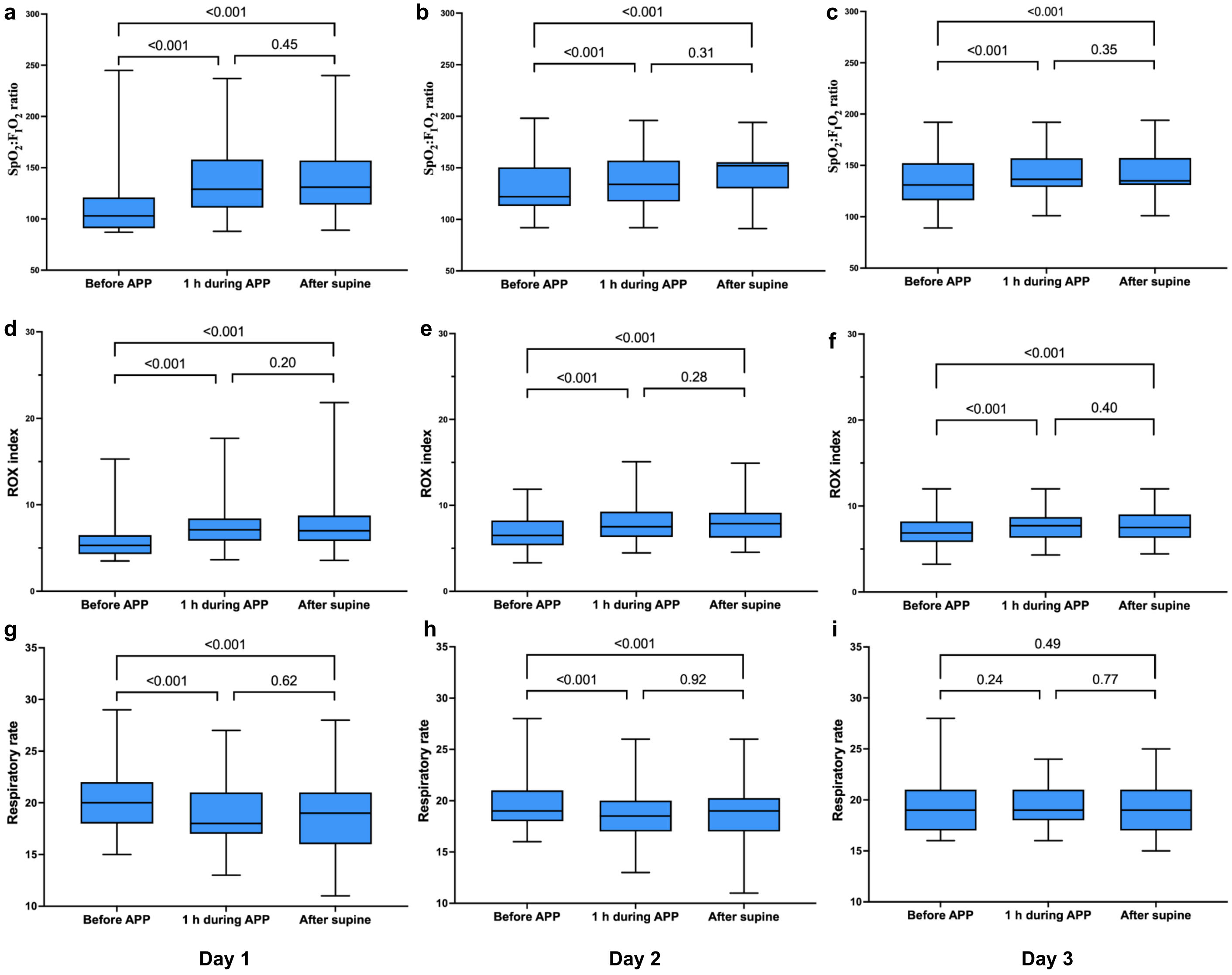

Figure S6. Correlation between response in LUS score with SpO_2:F_IO_2 ratio, ROX index and respiratory rate at the first morning APP sessions of the 1st (**a, b, c**), 2nd (**d, e , f**) and 3rd (**g, h, i**) days. LUS, lung ultrasound; APP, awake prone positioning; RR, respiratory rate

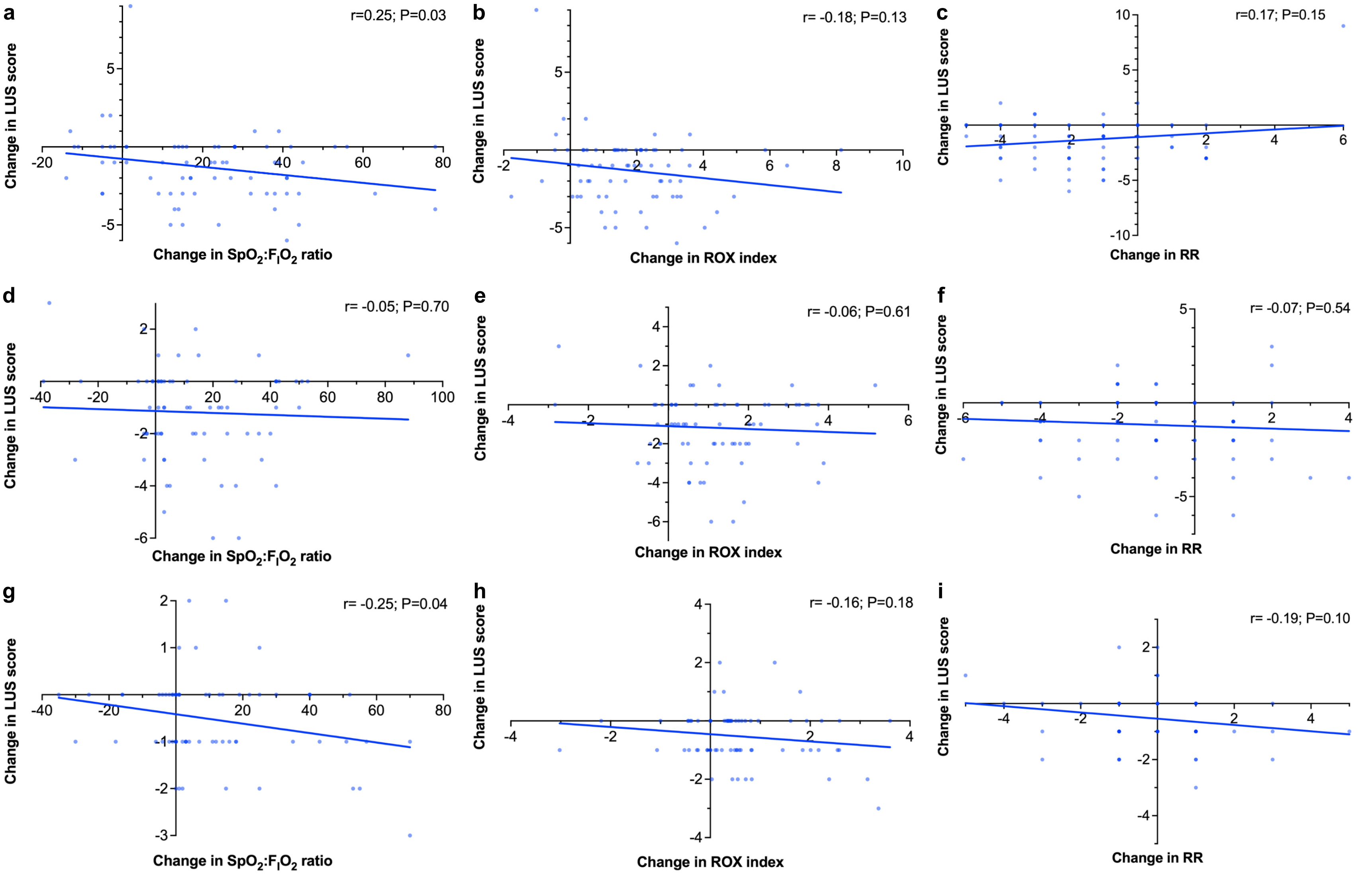

Figure S7. Comparison between areas under the ROC curves of responses to APP at the first session. ROC, receiver operating characteristic; APP, awake prone positioning.

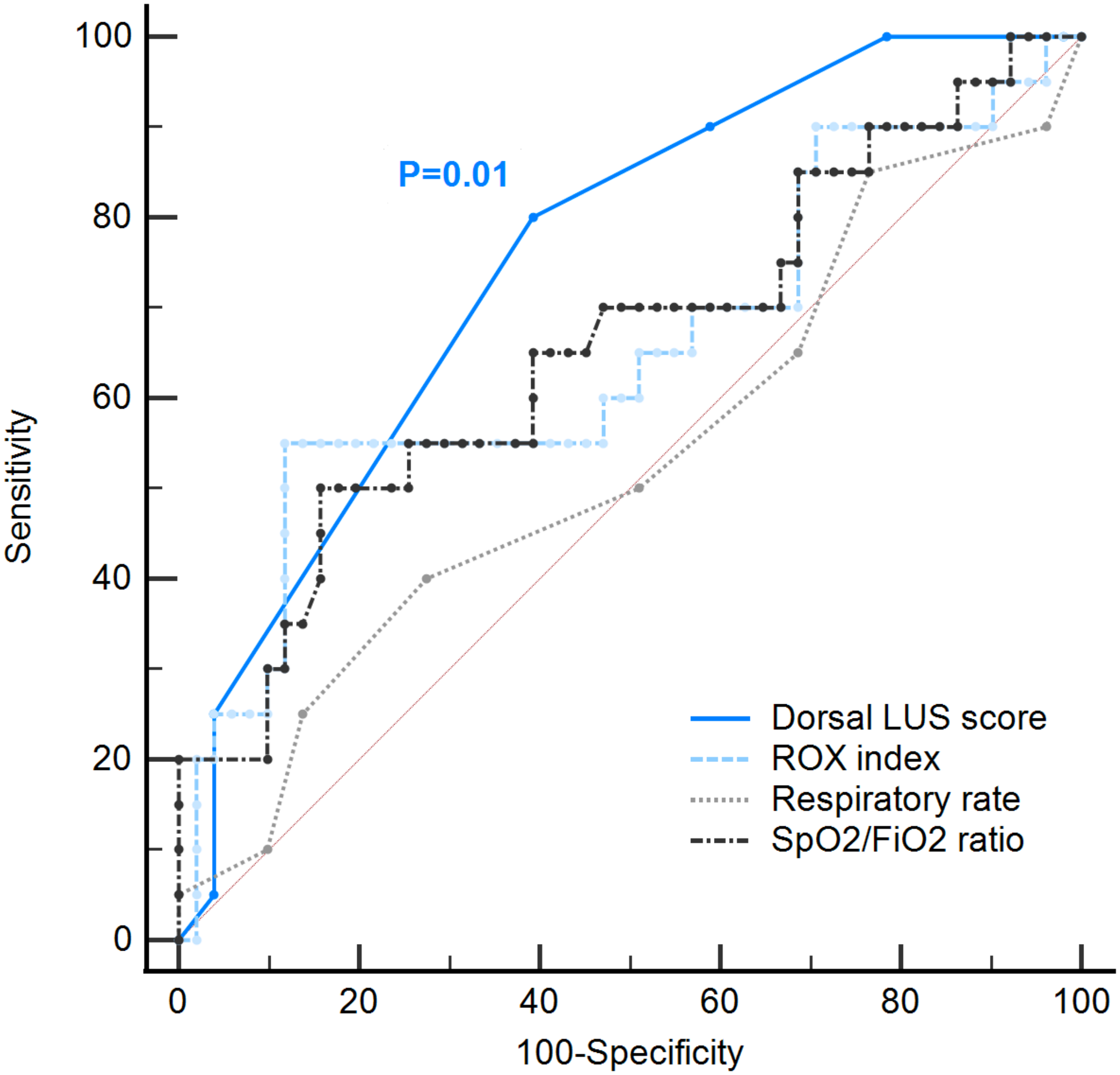

Table S2. Logistic regression to analyze the effect of ≥1 point decrease in dorsal LUS score at the first APP on the risk of intubation.

**Variable**	**Odds Ratio**	**95%CI**	**P value**
Unadjusted ∆dorsal LUS score < -1 point after return to supine at the first APP *	0.16	0.04-0.55	0.003
Adjusted by Age	0.20	0.05-0.71	0.01
Adjusted by days from COVID confirmation	0.14	0.04-0.53	0.003
Adjusted by pulmonary disease	0.16	0.04-0.59	0.005
Adjusted by chronic kidney disease	0.15	0.04-0.55	0.004
Adjusted by hypertension	0.15	0.04-0.54	0.003
Adjusted by baseline SpO_2:F_IO_2	0.16	0.04-0.55	0.003
Adjusted by **Δ**-dorsal LUS score after 1h on APP	0.14	0.03-0.56	0.005
Adjusted by **Δ**-SpO_2:F_IO_2 after 1h on APP (%)	0.16	0.04-0.58	0.004
Adjusted by **Δ**-SpO_2:F_IO_2 after return to supine (%)	0.19	0.05-0.68	0.01
Adjusted by **Δ**-RR after 1h on APP	0.16	0.04-0.57	0.04
Adjusted by **Δ**-RR after return to supine	0.16	0.04-0.55	0.003
Adjusted by **Δ**-ROX after 1h on APP	0.17	0.05-0.62	0.007
Adjusted by **Δ**-ROX after return to supine	0.16	0.04-0.59	0.005
Adjusted by mean daily APP duration at first 3 days	0.19	0.04-0.78	0.02

*All **Δ**-values are referred to the first session at the first day of recruitment
